# Extrinsic polarity cues control lamination versus cluster-based organization in vertebrate retinal development

**DOI:** 10.1016/j.isci.2026.115684

**Published:** 2026-04-11

**Authors:** Christina Schlagheck, Xenia Podlipensky, Cassian Afting, Ronald Curticean, Irene Wacker, Rasmus R. Schröder, Venera Weinhardt, Lucie Zilova, Joachim Wittbrodt

**Affiliations:** 1 Centre for Organismal Studies, Heidelberg University, Heidelberg, Germany; 2 Heidelberg International Biosciences Graduate School HBIGS, Heidelberg, Germany; 3 HeiKa Graduate School on “Functional Materials”, Heidelberg, Germany; 4 Bioquant, Heidelberg University, Heidelberg, Germany; 5 Institute of Microstructure Technology, Karlsruhe Institute of Technology, Karlsruhe, Germany

**Keywords:** biological sciences, cell biology, developmental biology

## Abstract

A wide range of retinal architectures allow metazoans to sense and respond to their environment. Early eye anlagen in both invertebrates and vertebrates share an initial pseudo-stratified epithelial organization, which in vertebrates forms a laminated retina, whereas invertebrate neuroepithelia differentiate into repetitive clusters. The contribution of epithelial polarity to vertebrate retinal lamination has remained difficult to address *in vivo*. Using medaka (*Oryzias latipes*) retinal organoids, we modulated polarity to assess its impact on the retinal architecture. We show that organoids undergo a polarity-dependent morphological switch: continuous apico-basal cues promote the formation of laminated retinal epithelia, whereas their loss induces horizontally organized clusters of retinal cells constituting retinal columns. The addition of laminin restores lamination and switches the clustered architecture back to the familiar vertebrate organization. Our findings reveal unexpected plasticity in vertebrate retinal development and uncover an alternative mode of retinal patterning emerging in the absence of extrinsic polarity signals.

## Highlights

• Differentiating retinal cells form clusters in medaka retinal organoids
• Retinal cell clusters include cell types of the retinal column in a recurrent ratio
• Retinal cell clusters arrange in periodic manner in the organoid cortex
• Supply of laminin switches clustered to laminated cell arrangement

## Graphical abstract

**Figure:**
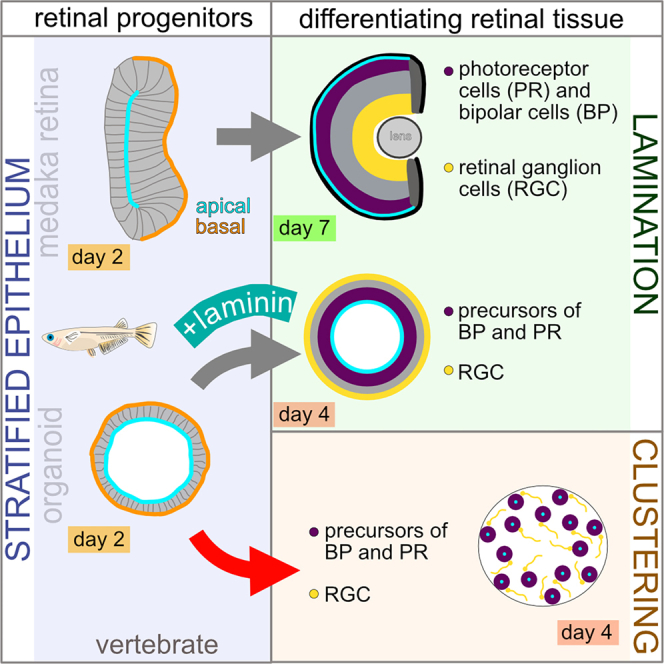

## Introduction

Photosensitive organs are essential for most animals to perceive and respond to their environment. While the gene regulatory networks establishing retinal identity are deeply conserved across metazoans,^1^^,^^2^^,^^3^ the retinal architecture varies widely—from invertebrate compound eyes to vertebrate camera-type eyes.^4^^,^^5^^,^^6^

Despite this morphological diversity, early eye anlagen in both invertebrates and vertebrates share an initial pseudo-stratified epithelial organization,^7^^,^^8^^,^^9^^,^^10^ which is maintained and elaborated into multilayered retinae in vertebrates. In contrast, the invertebrate neuroepithelium is re-organized as the ommatidia develop. Laminar organization of the vertebrate retina appears to be a consequence of initial polarization of the retinal neuroepithelium. This is, however, challenging to test in the organismal context.

Studies on disrupted polarity in the zebrafish photoreceptor (PR) layer have revealed a disturbed lamination despite ongoing differentiation.^11^^,^^12^^,^^13^ Perturbation of N-cadherin yields PR rosettes that later polarize locally, rather than assembling a continuous outer nuclear layer, when apically polarizing cues are lost.^14^ Studies on self-organization of dissociated neural tissues have shown that during re-aggregation, differentiated cells assemble into rosette-like microarchitectures.^15^^,^^16^

To address the plasticity of the forming retinal architecture and the impact of epithelial polarity on the differentiation and formation of retinal architecture, we take advantage of retinal organoids derived from medaka (*Oryzias latipes*),^17^ which allow us to specifically modulate polarity cues and test their impact on the level of epithelialization and structural organization of the forming retina.

We show that under specific culture conditions, medaka retinal organoids undergo a striking morphological switch depending on the level of apico-basal polarity imposed. When polarity cues are continuously provided, a laminated retinal epithelium is established in the organoid. The absence of polarity cues results in the formation of horizontal cellular clusters containing the retinal cell types, which form the vertical retinal column in the developing embryo.

We demonstrate that the emergence of this alternative retinal architecture is associated with a loss of epithelial polarity, notably the absence of extracellular matrix (ECM) components, such as laminin, which efficiently rescues lamination. Our findings indicate that tissue-level polarization and lamination in vertebrate retinae require specific extrinsic cues and that in their absence, differentiating retinal cell types self-organize into structurally distinct, retinal units. This reveals an unexpected plasticity in vertebrate retinal development and indicates a potential for alternative modes of retinal patterning.

## Results

### Retinal cell types in maturing retinal organoids emerge in spatially clustered domains

**Figure 1:**
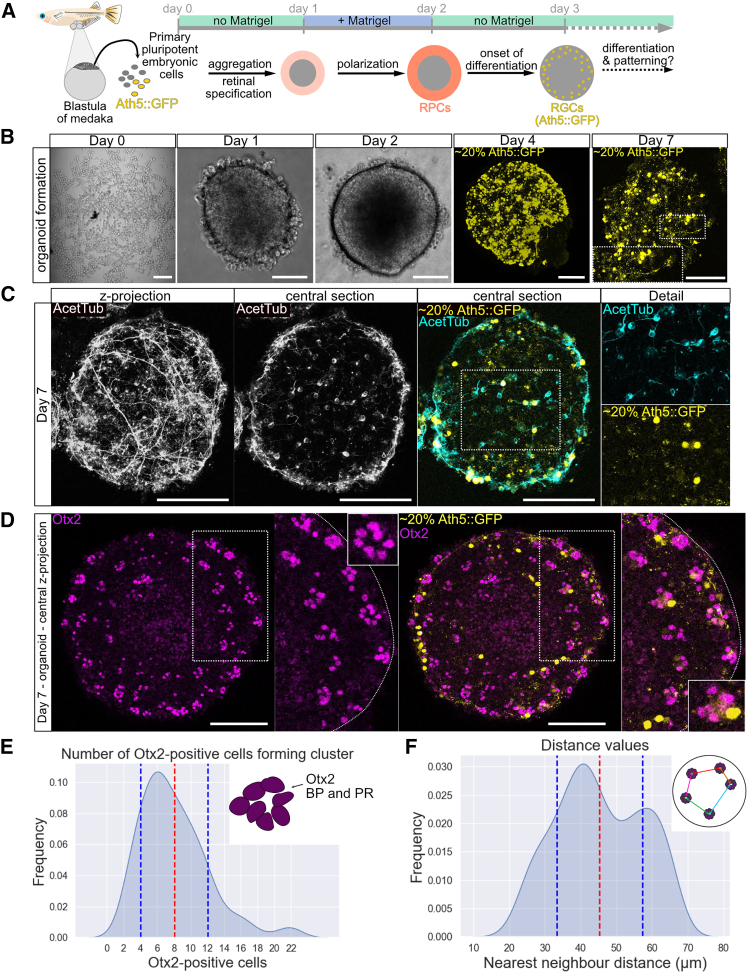
Neural retina in late medaka retinal organoids is structured in clusters (A) Scheme of retinal tissue development in medaka retinal organoids, as shown previously.^17^ Neural retinal (NR) development of retinal organoids depicted from blastula stage (day 0), highlighting retinal tissue specification. Primary embryonic cells were isolated from blastula-staged medaka embryos and seeded for aggregation. Integrating cells from transgenic blastulas carrying a construct marking RGCs (Ath5::GFP) in the cell suspension (20%) enabled tracing of RGCs. Aggregation and retinal specification were established by day 1 (red). Matrigel was added to the organoid culture on day 1, and by day 2, RPCs were formed in the cortex of the organoid sphere (orange). More centrally (gray), the organoid contains non-retinal cells. NR cell differentiation was initiated by day 3 with the onset of RGC differentiation (yellow). (B) Representative images of organoids over the course of seven days. Single cells in culture wells on day 0 (scale bar is 200 μm) and the aggregated organoid on day 1. By day 4, RGC (Ath5::GFP) expression indicated NR fate of the organoid. By day 7, RGCs were maintained and visibly formed axonal projections throughout the organoid. Zoomed-on day 7 organoid image shows RGCs with an axon projecting centrally. For day 4 and day 7, maximum Z projections of the Ath5::GFP label are shown, illustrating the distribution of labeled RGCs (∼20% of RGCs) across the half sphere (proximal pole to equator) of the organoid. Scale bars for images depicting day 1 to day 7 are 100 μm. (C) Immunostaining for acetylated tubulin (AcetTub) marking axons in the retinal organoid at day 7. Axonal projections are visible in maximum-intensity Z projection. Central section showing single neurons in overlay with the RGC marker Ath5::GFP. Due to chimeric organoids carrying the Ath5::GFP label in ∼20% of RGCs, the AcetTub label displays all RGCs in NR of the organoid. Scale bars are 100 μm. (D) Otx2-positive cells (magenta) in the day 7 retinal organoid form clusters in the cortex of the organoid. Maximum-intensity Z projection of central equatorial slices of the organoid shows the distribution of Otx2-positive cells marking PRs, BPs, and RGCs (Ath5::GFP). RGCs localize adjacent to Otx2-positive cell clusters. Scale bars are 100 μm. (E) Quantification of overall cell number in Otx2-positive clusters in day 7 organoids. Frequency of cell counts is plotted relative to Otx2-positive cell count (kernel density estimate (KDE) plot). The number of cells within one cluster ranges from 2 to 22, the mean value is marked by red dashed line, and standard deviation is indicated by blue dashed lines. *n* = 166 clusters were counted manually across *n* = 18 organoids. (F) Distance between the retinal cell clusters in direct neighborhood. Frequency of distance values between neighboring clusters plotted relative to the distance in μm (KDE plot). Mean distance to the nearest neighbor is 45.34 μm (organoids: *n* = 10 from 2 independent experiments; clusters, *n* = 159; nearest neighbor distances, *n* = 279). See also Figure S1.

Medaka retinal organoids provide a rapid and tractable system to interrogate tissue patterning and polarity in a vertebrate context. Under previously established conditions,^17^ aggregates of blastula-stage, pluripotent cells reproducibly adopt retinal identity already at day one of culture (Figures 1A and 1B). Providing a basal polarity cue via Matrigel at this stage resulted in epithelialization and the formation of a continuous layer of multipotent retinal progenitor cells (RPCs) by day two (Figures 1A and 1B).

From this organized neuroepithelium of RPCs, major neuroretinal cell types—including retinal ganglion cells (RGCs) and precursors of bipolar (BP) cells, PRs, and horizontal cells (HCs)^17^—differentiated over the next days in the absence of any further exogenous growth factors or patterning cues (Figures 1A and 1B).

To define the cellular repertoire and spatial arrangement of retinal cell types generated in late organoids, we analyzed day seven retinal organoids, corresponding to the developmental stage at which the embryonic medaka retina reaches full lamination (stage 39).^10^^,^^18^

We first focused on RGCs, the earliest-born retinal neurons.^10^^,^^19^ Chimeric organoids generated by the co-aggregation of wild-type cells with Ath5::GFP reporter cells^17^^,^^20^ labeled ∼20% of RGCs, facilitating the dynamic visualization of RGC emergence and distribution.

GFP-positive RGCs appeared consistently by day four (Figures 1B and S1), mirroring embryonic timing.^10^^,^^17^^,^^20^ Axonal projections were found from day four and maintained through day seven (Figures 1B and S1). Immunostaining of day seven organoids for acetylated tubulin highlighted axonal projections spanning both cortical and central regions of the organoid (Figure 1C). Overlay with Ath5::GFP confirmed label co-localization and indicated a broad distribution of RGCs throughout the organoid (Figure 1C). Together, these data establish that medaka retinal organoids faithfully recapitulate temporal neurogenesis and generate spatially distributed RGCs that extend axonal projections without external patterning inputs.

We next assessed the distribution of PR and BP cells by Otx2 immunostaining. Strikingly, Otx2-positive cells were not arranged in laminated layers in the cortex of the retinal organoid. Instead, they were arranged into recurrent multicellular clusters, tightly associated with RGCs (Ath5::GFP) (Figure 1D). Quantitative analysis revealed that the clusters stereotypically contained eight cells on average (Figure 1E). 3D reconstruction further demonstrated that the clusters were evenly spaced within the cortical domain of the organoid, with nearest-neighbor distances displaying a bimodal distribution and an average spacing of ∼45 μm (Figure 1F). Altogether, these observations revealed that in the absence of extrinsic patterning and polarity cues, retinal cells in organoids self-organize into repeating, stereotypically sized and spaced clusters.

### Retinal clusters represent a structural unit with stereotypic composition

The observation that BP-PR co-positive cells occur in discrete clusters suggested that retinal neurons in organoids assemble in clustered, horizontal units of 25 μm height, rather than in the vertical, columnar arrangement, characteristic for the stage-matched vertebrate retina (100 μm). To determine whether these organoid-derived clusters represent a re-arranged version of the canonical retinal column, we assessed the cellular composition and 3D organization of cells within each unit.

**Figure 2:**
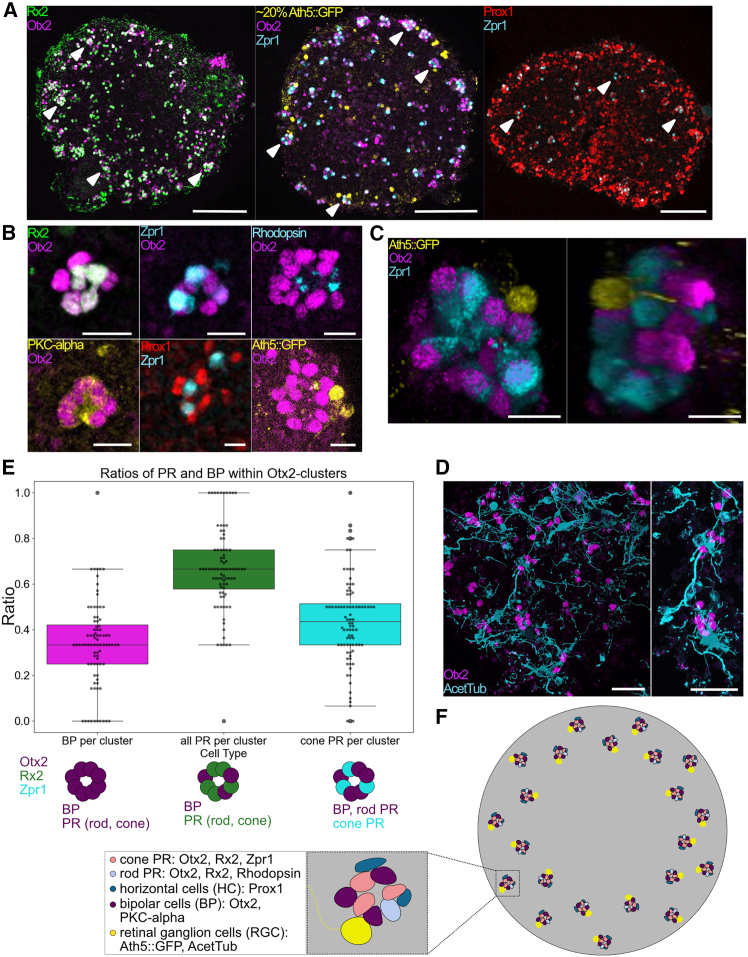
Retinal cell clusters include most neuroretinal cell types and form a recurrent structural unit in medaka retinal organoids (A) Retinal cell marker for photoreceptors (PRs) (Rx2), cone PRs (Zpr1), horizontal cells (HCs) (Prox1), retinal ganglion cells (RGCs) (Ath5::GFP), and the common marker for BPs and PRs (Otx2) across central sections of day 7 organoids. Filled white arrow heads mark examples of clusters with cell types represented. Scale bars are 100 μm. (B) Differentiated retinal cell types within and adjacent to Otx2-positive cell clusters. Otx2-Rx2-double-positive cells mark PRs (*n* = 16 organoids in 3 independent experiments) within cell clusters, and Otx2-positive Rx2-negative cells mark BP cells. PKC-α marking BP specifically (*n* = 6 organoids in 2 independent experiments). Otx2-Zpr1-positive cells represent cone PRs (*n* = 31 organoids in 6 independent experiments), and Otx2-Rhodopsin-positive cells mark rod PRs (*n* = 15 in 3 independent experiments). Prox1-positive cells mark HCs (*n* = 16 organoids in 4 independent experiments) adjacent to Zpr1-positive cone PRs. Scale bars are 10 μm. (C) 3D reconstruction of a single cluster labeled for Otx2, Zpr1, and Ath5::GFP. 3D-reconstructed cluster is shown from two angles, rotated by 100°. Scale bars are 10 μm. (D) Immunostaining for Otx2 and acetylated tubulin markers showed the association of Otx2-labeled cell clusters with neurons. Axonal projections span between clusters. Scale bars are 25 μm. (E) Comparison of the relative contribution of BPs, all PRs, and cone PRs within cell clusters. Boxplots showing ratios of the numbers of BPs to the number of Otx2-positive cells per cluster (BP/Otx2-positive cluster) (median = 0.33), all PRs/Otx2-positive cluster (median = 0.67), and cone PRs/Otx2-positive cluster (median = 0.44). All PRs were quantified by manually counting the number of Rx2-Otx2-double-positive cells within an Otx2-positive cell cluster, and from the same datasets, BPs were scored as Otx2-only cells. Within *n* = 9 organoids from 2 independent experiments, *n* = 78 clusters were quantified. From different samples, Zpr1-Otx2-double-positive cells were scored against Otx2-positive cell count within respective clusters. For *n* = 9 organoids from 3 independent experiments, *n* = 88 clusters were quantified manually. (F) Schematic summary of retinal cell types identified within retinal organoids at day 7 and their respective labels and positional relation. See also Figures S2 and S3 and Video S1.

To identify which retinal neurons differentiated in the organoids, we performed immunostaining for cell type-specific markers verified based on stage-matched medaka retinal sections (Figure S2). Otx2-Rx2-double-positive PRs were detected in every cluster (Figures 2A and 2B). We identified both cone PRs (Zpr1) and rod PRs (rhodopsin) among the PR cells within the Otx2-positive clusters (Figures 2A and 2B).^10^ HCs, characterized by the expression of Prox1,^21^ localized in close proximity to the cone PR marker (Figures 2A and 2B). BP cells were identified among the Otx2-positive cells by co-staining for PKC-α^22^; therefore, BPs were localized next to PRs (Figure 2B). RGCs were consistently juxtaposed to the clusters (Figures 2A and 2C). Strikingly, we found that by day seven, all traceable neural retinal neurons (5 out of 6) differentiated in the organoids and were all either contained in or tightly associated with the Otx2-positive clusters described above (Figures 2A and 2B). Ultrastructural analysis by scanning electron microscopy confirmed tight cell-cell contacts within each cluster, as well as the presence of cilia, a hallmark of maturing PRs (Figure S3 and Video S1).^10^^,^^23^

Video S1. Extending cilia from cells within the retinal cluster visualized with electron microscopy, related to Figures 2 and S3 Cilium 1 (red) and cilium 2 (green) are outlined in the image stack. Scale bars are 5 μm.

In the vertebrate (fish) retina, PRs, BPs, and RGCs are arranged in distinct layers connected vertically via synaptic circuits to propagate visual information. In contrast, in organoids under the conditions described, these same cell types self-assembled into compact clusters, with PRs, BPs, and RGCs located in direct apposition (Figures 2A–2C). The cellular composition was not only limited to a single cluster but appeared recurrently across the organoid (Figure 2A). This was further validated by the quantitative analysis, which revealed a stereotypic cellular composition. On average, Otx2-positive clusters consisted of ∼67% PRs and ∼33% BPs, with cones comprising two-thirds of the PR population (∼44% of all Otx2-positive cells) (Figure 2D). This regularity was consistent across organoids, underscoring a recurrent “patterning logic” in which clusters combine the major cell types in reproducible ratios (Figure 2F). Complementary labeling with acetylated tubulin confirmed the regular apposition of these clusters to axon-bearing neurons, and tubulin-positive projections extended between clusters, hinting at emerging connectivity across units (Figure 2E).

These data establish that retinal cells in vertebrate organoids organize into discrete, reproducibly composed and distributed clusters that contain all elements establishing the retinal column as found in the maturing medaka retina at comparable stage. The emergence of such clusters stands in stark contrast to the canonical, layered architecture of vertebrate retinae.

### Reorganization of neural retinal tissue goes along with loss of epithelialization

During embryonic development of the medaka retina, proliferation, differentiation, and positioning of neuronal subtypes occur in a temporally ordered and spatially coordinated manner, emerging from a common pool of RPCs.^10^ Cellular ratios essential for retinal function are thought to be maintained either through lineage relationships or by signaling across neighboring progenitors.^24^^,^^25^^,^^26^^,^^27^ In later stages, studies on post-embryonic growth have shown that the full complement of retinal cell types arises from single stem cells, underscoring their intrinsic multipotency.^28^

**Figure 3:**
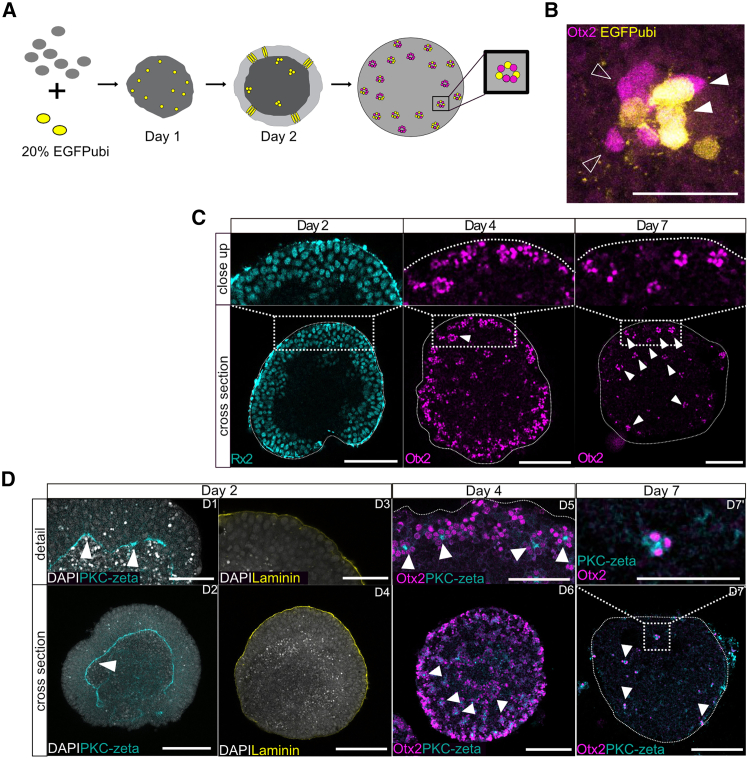
Progressive retinal cell cluster formation along with the loss of epithelial structure in medaka retinal organoids (A) Scheme for the experimental setup of clonal tracing in the medaka retinal organoid, and a schematic representation of the outcome. Unlabeled wild-type cells were mixed with GFP-labelled cells while seeding organoids on day 0. Over the course of maturation, GFP-positive clones underwent expansion. Analyzing GFP-labeled and unlabeled cells within Otx2-positive clusters allowed to follow-up on the clonal relationships of cells within a retinal cell cluster. (B) Otx2-expressing clusters were composed of cells of different clonal origins, as seen in the mix of GFP-labelled cells (filled arrow head) and GFP-negative cells (empty arrow head). Scale bar is 25 μm. (C) Retinal organoids stained for the cellular distribution of retinal cells marked by Rx2 (RPCs) (day 2) or Otx2 on day 4 (*n* = 100 organoids in 14 independent experiments) and day 7 (*n* = 75 organoids in 11 independent experiments). Otx2-expressing cell clusters are indicated by white filled arrowheads. Zoom on a central slice showing a cortical section in detail. Scale bars are 100 μm. (D) Polarity of retinal tissue in medaka retinal organoids from day 2 to day 7. Optical sections of organoids are shown, with PKC-zeta (PKC-ζ) marking the apical side, and laminin marking the basal side of cells. Polarity of RPCs on day 2 in retinal organoids shows global structuring as an epithelium, with the apical (PKC-ζ) marker on the inner surface of the epithelium (marked by arrow head) and basal marker (laminin) on the periphery. By day 4, Otx2-positive clusters formed in the organoid, with the apical side toward the center of the cluster (marked by arrow heads). By day 7, the polarity was retained as the apical marker PKC-ζ can be found in the center of Otx2-positive clusters. Scale bars are 100 μm (organoid cross-sections) and 50 μm (organoid detail). See also Figures S4 and S5.

To address the emergence of the retinal cell clusters, we assessed the origin of the cells within a cluster, either from a single progenitor or multiple progenitors. We performed clonal analysis in a chimeric assay by mixing blastula cells ubiquitously expressing GFP (20% EGFPubi) with unlabeled wild-type cells while seeding (Figure 3A). Lineage tracing revealed that all Otx2-positive clusters invariably contained both labeled and unlabeled cells (Figure 3B). This indicated that clustering is not the result of clonal expansion but rather reflects an active assembly. Thus, cells of different origins tend to assemble in the correct cell type ratios to form multicellular clusters.

We next followed the formation of clusters over time to define a temporal window relevant for the structural organization of the tissue. On day two, RPCs established a continuous epithelial sheet of cells organized in vertical orientation relative to the cortex plane. By day four, neural retinal cell differentiation was initiated with Otx2-positive PR and BP precursors emerging from and distributed predominantly at the organoid cortex (Figure 3C).^17^ At this stage, first indications of Otx2-positive cell clustering became apparent (Figure 3C). By day seven, all Otx2-positive cells had organized into discrete clusters, spaced at regular intervals across the organoid cortex (Figure 3C). Notably, the clustered phenotype persisted stably beyond day seven (day nine and day eleven; Figure S4), consistent with the establishment of a constructive and self-maintained architecture.

Cellular positioning of the differentiating cell types in the embryonic retina is oriented within the apico-basal axis of the neuroepithelium. The clustered organization in the retinal organoid suggests that the epithelial organization is affected. We, therefore, traced epithelial polarity over time by analyzing the distribution of apical and basal polarity markers in the retinal organoid tissue. We found that on day two, RPCs exhibited a coherent epithelial organization, with apical domains facing the organoid core and basal domains exposed at the surface, a pattern comparable to the optic cup *in vivo* (Figures 3D and S5). However, between day two and day four, this global epithelial organization was progressively lost, as seen by the distribution of differentiating retinal cells marked by Otx2. With the emergence of Otx2-positive cell clusters, the common apical side of the former epithelium was not detectable anymore by the apical polarity marker PKC-ζ. By day seven, the polarity of the tissue was reorganized to a local pattern: each cluster established its own apico-basal orientation, with apical domains directed toward the cluster center (Figure 3D). This local polarity pattern remained stable through subsequent stages. In contrast, the embryonic medaka retina maintained a continuous apico-basal polarity throughout development, with PRs localized apically and RGCs basally (Figure S5).

Thus, the transition from a continuous epithelium to discrete retinal clusters represents a structural re-organization unique to the *in vitro* environment and not observed in normal vertebrate retinal development. The loss of tissue-wide epithelialization suggests that external inputs—present in the embryonic context but absent in the artificial culture environment—are required to maintain the continuous epithelial organization necessary for layered retinal architecture.

### Laminin supports epithelial structure in retinal organoids

**Figure 4:**
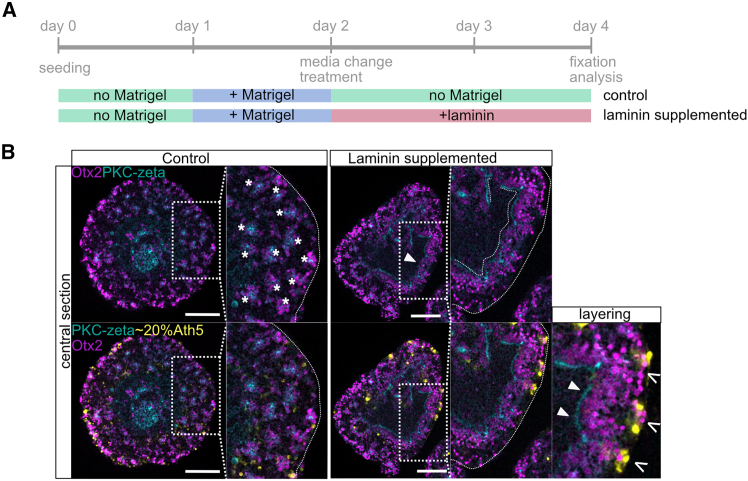
Supplementation of medaka retinal organoids with laminin is sufficient to restore epithelial layering of neurons in retinal organoids (A) Experimental outline of organoid culture supplemented with 25 μg/mL laminin from day 2 to day 4. Organoids were grown until late day 2 under standard conditions (control) with addition of 2% Matrigel on day 1. Preceding the loss of tissue polarity, the medium was changed; for laminin-exposed samples, 25 μg/mL laminin was added to the well. The samples were incubated until day 4. Afterward, the samples were fixed, immune stained, and imaged. (B) Loss of layering upon the progression of organoid development after day 2 could be partially prevented by laminin supplementation by day 2. In laminin-supplemented culture, tissue stretches stayed polarized by day 4, and cluster formation was prevented. Otx2-positive cells lined up with apical pole toward the center, and RGCs were arranged in an adjacent layer facing the outer rim of the organoid. In five independent experiments, out of *n* = 28 organoids, 24 (85%) showed tissue polarization after laminin treatment. Scale bars are 100 μm. See also Figure S6.

The establishment and maintenance of epithelial organization in the retina are closely linked to the ECM, with laminins representing a key component of retinal ECM.^29^^,^^30^ In our organoid system from day two onwards, cultures were maintained without ECM supplementation. To test whether external cues, such as laminin, are sufficient to preserve epithelial tissue architecture during the onset of retinal differentiation, we supplemented cultures with laminin (laminin-nidogen complex, Roche), starting at day two. This time point is prior to the breakdown of epithelial layering observed in standard conditions (Figure 4A). Indeed, organoids retained integrity of epithelia and layering of retinal cell types by day four when cultured with external addition of laminin (Figures 4B and S6). Importantly, tissue polarity was preserved, with the apical side of the epithelium oriented toward the organoid core. Thus, laminin provides the external structural cue that is absent in standard culture conditions and is sufficient to maintain epithelial tissue continuity. Within these organoids, Otx2-positive cells localized apically, while RGCs occupied the outer cortex, mirroring the relative arrangement of cell types in the embryonic retina.

The preservation of epithelial layering and the pseudo-stratified organization of retinal cell types as in the fish retina by the presence of laminin underscore the critical role of ECM in instructing epithelial tissue architecture and polarity. In contrast, organoids grown without laminin abandon epithelial layering and, instead, adopt a clustered organization of retinal cells. The process of epithelial disruption and retinal cell clustering seen in the retinal organoids marks a striking alternative retinal tissue architecture for a vertebrate retinal model.

## Discussion

Our study identifies laminin as a critical extracellular cue for maintaining epithelial organization in medaka retinal organoids.

When laminin is not provided exogenously, epithelial continuity is lost and retinal cells adopt a clustered, unit-based arrangement. Laminin supplementation preserves epithelial layering and polarity, resulting in a layered arrangement, with Otx2-positive cells oriented apically and RGCs basally, thereby resembling the vertical organization of a retinal column in the embryonic retina. These findings highlight the instructive role of ECM in organizing the retinal tissue architecture and uncover an alternative patterning mode that emerges when the epithelial integrity is not sustained.

Other vertebrate retinal organoid models based on mouse or human cells achieve a layered tissue organization without prolonged exogenous ECM exposure. This indicates that in mammalian retinal organoids in general and in human organoids in particular, cells provide laminin and other ECM components autonomously.^31^^,^^32^ The presence of laminin is required for proper lamination and differentiation.^30^ In medaka retinal organoids, in contrast, we showed that the laminar structuring of the retinal cell types depends on exogenous supplementation of laminin. This species-specific difference in self-sufficiency highlights how variations in ECM contribution bias tissue architecture toward layered versus modular outcomes. The clustered assemblies of retinal cells formed in laminin-free medaka organoid cultures suggest that, in the absence of epithelia-polarizing signals, retinal cells follow an intrinsically encoded or physically advantageous strategy, once tissue-wide epithelial continuity is not maintained.

RGCs, the first-born retinal cell type, could form a nucleation point for the forming retinal cell clusters.^33^ The recurrent inclusion of PRs, BPs adjacent to RGCs within these clusters, and spatial separation of the single units points to a regulated process faithfully assembling the essential components of the retinal column. This unit-based organization established along with retinal cell differentiation is reminiscent of ommatidial patterning in the *Drosophila* retina, where PR clusters arise as structurally and functionally independent modules. Ommatidia emerge from an initially polarized epithelium, with unit formation proceeding concomitantly with the establishment of the morphogenetic furrow and the perpendicular patterning axis.^34^ The resemblance of laminin-free retinal organoids to the unit-based architecture of the invertebrate eye suggests that the breakdown of epithelial organization may underlie divergent patterning strategies of retinae across the animal kingdom. In light of our observations, it is tempting to speculate that epithelial continuity represents a key decision point between layered and unit-based architectures of the retina.

**Figure 5:**
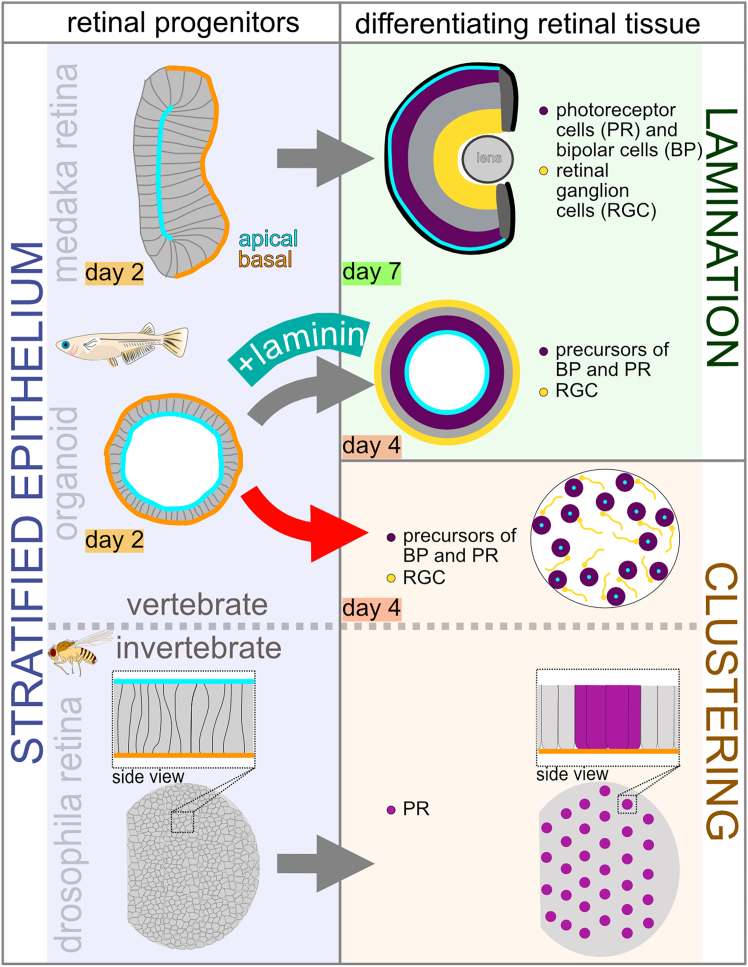
Proposed structuring similarities between vertebrates and invertebrates based on the epithelial vs. non-epithelial tissue organization during retinal cell differentiation

Taken together, our findings raise the possibility that the evolutionary divergence of vertebrate and invertebrate eye types reflects alternative solutions to the challenge of organizing PR circuits: continuous epithelial layering, supported by ECM, versus modular unit formation upon epithelial modulation. The medaka organoid system, in which both modes can be elicited, thus, provides the unique opportunity to dissect polarity-dependent retinal structuring and to experimentally explore potential evolutionary trajectories in eye design (Figure 5).

More generally, organoid systems reveal latent self-organizing capacities that are normally masked by embryonic constraints. By uncovering alternative tissue architectures, such as layered epithelia versus unit-based modules, organoids serve not only as models for development and disease but also as experimental windows into evolutionary processes and the fundamental principles of tissue patterning.

In this sense, retinal organoids offer a rare experimental system to replay alternative evolutionary scenarios of eye design in real time.

### Limitations of the study

Within this study, we describe the formation of retinal cell clusters in medaka organoids based on immunolabeling and a genetic reporter line. Further, more specific subpopulations of retinal cell types or the presence of amacrine cells were not addressed. To detail specific cell type ratios in clusters in relation to the corresponding distribution in the laminated retina, future studies will require prolonged culture of laminin-supplemented organoid culture and high-resolution molecular analysis such as single-cell RNA expression analysis. While there is a striking resemblance to invertebrate functional units, the functionality of the clusters identified awaits further experimental validation.

## Resource availability

### Lead contact

Requests for further information and resources should be directed to and will be fulfilled by the lead contact, Joachim Wittbrodt (jochen.wittbrodt@cos.uni-heidelberg.de).

### Materials availability

This study did not generate new unique reagents.

### Data and code availability

• All data reported in this paper will be shared by the lead contact upon request.
• This paper does not report original code.
• Any additional information required to reanalyze the data reported in this paper is available from the lead contact upon request.

## Acknowledgments

The authors thank the members of the Wittbrodt lab for critical feedback and support throughout the project. We are indebted to L. Centanin and G. Jékely for extensive and critical feedback on the manuscript. We thank the fish team (A. Sarazeno, M. Liv, and E. Leist) for excellent animal husbandry. This work was supported by grants of the Excellence Cluster “3D Matter Made to Order” (EXC-2082/1-390761711) funded through the German Excellence Strategy via 10.13039/501100001659Deutsche Forschungsgemeinschaft to J.W. and R.R.S. and of postdoc take-off grant funded by the Excellence Cluster “3D Matter Made to Order” to L.Z. and V.W.

## Author contributions

C.S. and J.W. designed the experimental strategy; C.S. performed the experiments and, in collaboration with X.P. and C.A., analyzed the data; R.C., I.W., and R.R.S. performed the EM analysis; C.S. created the display items with critical inputs from J.W., V.W. and L.Z.; C.S. and J.W. wrote the manuscript, with refined input from all co-authors.

## Declaration of interests

The authors declare no competing interests.

## STAR★Methods

### Key resources table

**Table:** 

REAGENT or RESOURCE	SOURCE	IDENTIFIER
**Antibodies**		

mouse anti-acetylated tubulin	Sigma Aldrich	Cat# T7461
chicken anti-GFP	Thermo Fisher Scientific	Cat# A10262; RRID:AB_2534023
mouse anti-HuC/D	Thermo Fisher Scientific	Cat# A21271; RRID:AB_221448
rabbit anti-Laminin	Sigma-Aldrich	Cat# L9393; RRID:AB_477163
goat anti-Otx2	R&D systems	Cat# AF1979; RRID:AB_2157172
rabbit anti-PKC-alpha C-20	Santa Cruz	Cat# sc-208; RRID:AB_2168668
rabbit anti-PKC-zeta C-20	Santa Cruz	Cat# sc-216; RRID:AB_2300359
rabbit anti-Prox1	Millipore	Cat# AB5475; RRID:AB_177485
mouse anti-Rhodopsin	Sigma-Aldrich	Cat# MABN15
rabbit anti-Rx2	Reinhardt et al.^35^	N/A
mouse anti-Zpr1	Zebrafish International Resource Center	N/A

**Chemicals, peptides, and recombinant proteins**		

Aceton	Sigma Aldrich	Cat# 32201-2.5L
benzyldimethylamine (BDMA)	Serva	N/A
β-mercaptoethanol	Gibco	Cat# 21985023
BSA	Sigma Aldrich	Cat# A9418
DMEM/F12	Gibco,	Cat# 21041025
dodecenylsuccinic acid anhydride (DDSA)	Serva	Cat# 20755.01
fetal bovine serum	Sigma Aldrich	Cat# 12103C
glutaraldehyde	Plano	Cat# R1010
glycerol	Sigma Aldrich	Cat# V900122
glycid ether 100	Serva	Cat# 21045.01
HEPES pH 7.4	Carl Roth	Cat# 7365-45-9
knockout serum replacement (KSR)	Gibco	Cat# 10828028
laminin	Roche	Cat# 11243217001
lead citrate	Science Services	Cat# DM22410
Matrigel	Corning	Cat# 356230
MEM non-essential amino acids	Sigma Aldrich	Cat# M7145
methyl nadic anhydride (MNA)	Serva	Cat# 29452.01
N2 supplement	Gibco	Cat# 17502048
osmium tetroxide (OsO_4_)	Science Services	Cat# E19152
Paraformaldehyde (PFA)	Sigma Aldrich	Cat# P46148
PFA (for EM)	Plano	Cat# R1026
PBS	Thermo Scientific	Cat# 100110023
penicillin-streptomycin	Gibco	Cat# 15140122
PIPES buffer (pH 6.8.)	Carl Roth	Cat# 9156.3
potassium ferricyanide (K_3_[Fe(CN)_6_])	Science Services	N/A
sodium pyruvate	Sigma Aldrich	Cat# S8636
taurine	Sigma Aldrich	Cat# T8691
Tissue Freezing Medium	Leica	Cat# 1402018926
uranyl acetate	Science Services	Cat# E22400

**Experimental models: Organisms/strains**		

Medaka Cab (wild type)	Loosli et al.^36^	N/A
Medaka Ath5:GFP	Del Bene et al.^20^	N/A
Medaka Wimbledon dsTrap#6	Centanin et al.^28^	N/A

**Software and algorithms**		

Amira	Thermo Fisher Scientific	N/A
Atlas 5	Carl Zeiss Microscopy	N/A
Fiji (ImageJ2)	v. 2.16.0/1.54m	https://imagej.net/ij/
Jupyter lab	v. 4.4.0a1 – packages used: statannotations, seaborn, pandas, matplotlib, numpy	https://jupyter.org/install

### Experimental model and study participant details

#### Fish husbandry

Medaka (*Oryzias latipes*) stocks were maintained at 28°C in closed stocks with a 14 h light and 10 h dark cycle. Husbandry of the fish (permit number: AZ35-9185.64/BH; line generation permit: 35–9185.81/G-145/15 Wittbrodt) was performed according to the EU directive 2010/63/EU guidelines and the German animal welfare laws (Tierschutzgesetz §11, Abs. 1, Nr.1). Fish lines used were Cab^36^ as wild type, Ath5::GFP^20^ and Wimbledon dsTrap#6 line^28^ here called EGFPubi.

#### Medaka retinal organoid generation

Medaka organoids were prepared from late blastula stage primary embryonic stem cells as described in Zilova et al.^17^ Cells were extracted from stage 10^18^ by dechorionating embryos using hatching enzyme. The cell mass was manually detached from the yolk, washed and dissociated in 1× PBS (Thermo Scientific, Cat# 100110023). The cell suspension was strained by centrifugation for 3 min at 180×*g* and re-suspended in ‘differentiation medium’ (DMEM/F12 (Gibco, Cat# 21041025), 5% knockout serum replacement (KSR) (Gibco, Cat# 10828028), 0.1 mM MEM non-essential amino acids (Sigma Aldrich, Cat# M7145), 0.1 mM sodium pyruvate (Sigma Aldrich, Cat# S8636), 0.1 mM β-mercaptoethanol (Gibco, Cat# 21985023), 2 mM HEPES pH 7.4 (Carl Roth, Cat# 7365-45-9), 100 U mL^−1^ penicillin-streptomycin (Gibco, Cat# 15140122)). Per single organoid, 1500 cells in 100 μL were seeded into individual wells of a low binding U-bottom-shaped 96-well plate (Nunclon Sphera U-Shaped Bottom Microplate, Thermo Fisher Scientific, Cat# 174925). Cells of different genetic backgrounds were combined when indicated (20% Ath5GFP with 80% Cab cells; 20% dsTrap#6/ubiGFP with 80% Cab cells). The culture was incubated at 26°C without CO_2_ control over night (o.n.) and organoids were washed in differentiation medium early day 1. Under the standard conditions, day 1 organoids were supplemented with Matrigel (Corning, Cat# 356230) to a final concentration of 2%. From that timepoint on, organoids were incubated at 26°C with 5% CO_2_. On day 2, organoids were transferred into ‘maturation medium’ (DMEM/F12 (Gibco, Cat# 21041025), 10% fetal bovine serum (Sigma Aldrich, Cat# 12103C), 1× N2 supplement (Gibco, Cat# 17502048), 1 mM taurine (Sigma Aldrich,Cat# T8691), 20 mM HEPES pH 7.4 (Carl Roth, Cat# 7365-45-9), 100 U mL^−1^ penicillin streptomycin (Gibco, Cat# 15140122)) and media was changed on day 4 and day 7 for fresh maturation media.

### Method details

#### Supplementation of laminin to organoid culture

For supplementation of organoids with laminin from day 2, laminin protein solution (Roche, Cat# 11243217001, laminin-nidogen complex (1:1, higher than 90% purity tested by SDS page) from mouse Engelbreth-Holm-Swarm sarcoma)) was added to a final concentration of 25 μg/mL to one organoid per well. For the control condition, standard culture was performed as described above. Organoids were incubated at 26°C with 5% CO_2_ control until day 4.

#### Whole mount staining of medaka organoids

Organoids were fixed in 4% PFA (paraformaldehyde) (Sigma Aldrich, Cat# P46148) in 1× PTW (1× PBS, 0.05% Tween 20) for 48 h at 4°C. Samples were washed with 1× PTW and permeabilized with Aceton (Sigma Aldrich, Cat# 32201-2.5l) at −20°C for 15 min. Organoids were washed in 1xPTW and blocked in 10% BSA (Sigma Aldrich, Cat# A9418) in 1xPTW for 2 h at r.t. Primary antibodies (ABs) (mouse anti-acetylated tubulin (Sigma Aldrich, Cat# mA1-12717); chicken anti-GFP (Thermo Fisher Scientific, Cat# A10262); rabbit anti-Laminin (Sigma-Aldrich, Cat# L9393); goat anti-Otx2 (R&D systems, Cat# AF1979); rabbit anti-PKC-alpha C-20 (Santa Cruz, Cat# sc-208); rabbit anti-PKC-zeta C-20 (Santa Cruz, Cat# sc-216); rabbit anti-Prox1 (Millipore, Cat# AB5475); mouse anti-Rhodopsin (Sigma-Aldrich, Cat# MABN15); rabbit anti-Rx2 (Reinhardt et al., 2015); mouse anti-Zpr1 (Zebrafish International Resource Center)) were applied in 1% BSA in 1× PTW and incubated for 48 h at 4°C. Samples were washed in 1× PTW and the secondary ABs (Invitrogen) and DAPI (1:500) were applied in 1% BSA in 1× PTW o.n. at 4°C in darkness. Afterward, samples were washed in 1× PTW. For confocal microscopy, organoids were mounted in optical clearing solution.^37^

#### Cryosectioning and immune staining of hatchlings

Medaka hatchlings (0 days post hatch) were anaesthesized and fixed in 4% PFA in 1xPTW for 48 h at 4°C. Heads were manually dissected using forceps. Samples were cryopreserved in 30% (w/w) sucrose and sectioned to 16 μm sections in Tissue Freezing Medium (Leica, Cat# 1402018926). Sections were rehydrated with 1× PTW for 10 min and blocked with 10% BSA in 1× PTW for 2 h in a humidified chamber. After washing in 1× PTW, primary ABs (anti-GFP chicken (Thermo Fisher Scientific, Cat# A10262); anti-HuC/D mouse (Thermo Fisher Scientific, Cat# A21271), anti-Laminin rabbit (Sigma-Aldrich, Cat# L9393) with reactivity against laminin α-1; anti-Otx2 goat (R&D systems, Cat# AF1979); anti-PKC-alpha C-20 rabbit (Santa Cruz, Cat# sc-208); anti-PKC-zeta C-20 rabbit (Santa Cruz, Cat# sc-216); anti-Prox1 rabbit (Millipore, Cat# AB5475); anti-Rhodopsin mouse (Sigma-Aldrich, Cat# MABN15); anti-Rx2 rabbit (Reinhardt et al., 2015); anti-Zpr1 mouse (Zebrafish International Resource Center)) were applied in 1% BSA o/n at 4°C in a humidified chamber. Sections were washed in 1× PTW and secondary ABs (Invitrogen) and DAPI (1:500) were applied in 1% BSA in 1× PTW and incubated for 2 h at 37°C in a humidified chamber. Afterward, samples were washed in 1× PTW and mounted by adding 60% glycerol (Sigma Aldrich, Cat# V900122).

#### Light microscopy imaging and image processing

Live imaging of organoids was done using a ACQUIFER (ACQUIFER Imaging GmbH) imaging machine. Fixed organoid and embryo samples were imaged using a Leica TCS Sp8 Dmi8 inverted confocal microscope (20× and 63× oil immersion objective). For display, confocal images were subjected to rolling ball background subtraction of 25 or 50 pixels and median filtering (1.2 pixels) using the Fiji software. Within one condition, all images were treated the same way.

#### EM sample preparation and ultramicrotomy

Organoids (day seven) were fixed in 0.1 M PIPES buffer (pH 6.8.) (Carl Roth, Cat# 9156.3) + 1.25% glutaraldehyde (Plano, Cat# R1010) + 2% PFA (Plano, Cat# R1026) for several days. After washing with 0.1 M PIPES, the samples were incubated in 1% osmium tetroxide (OsO_4_) (Science Services, Cat# E19152) and 0.8% potassium ferricyanide (K_3_[Fe(CN)_6_]) (Science Services) for 1 h on ice. Subsequently, the samples were stained overnight with 2% uranyl acetate (Science Services, Cat# E22400) in 25% ethanol/water.

Samples were then subjected to a graded ethanol dehydration series (25%, 50%, 70%, 90%, and 100% ethanol/water), followed by ethanol/acetone (1:1) and 100% acetone for 15 min/dilution step. Samples were then infiltrated with Epon resin (42.4 g glycid ether 100 (Serva, Cat# 21045.01), 29.6 g dodecenylsuccinic acid anhydride (DDSA) (Serva, Cat# 20755.01), 18.4 g methyl nadic anhydride (MNA) (Serva, Cat# 29452.01), 2.4 g benzyldimethylamine (BDMA) (Serva) as initiator) first with 30% acetone, then with 70% Epon in acetone, each step for 2 h. Embedding of samples was done in 100% Epon resin. Polymerization was done at 60°C for 2 days.

Serial sections (100 nm thickness) were prepared using a PowerTome Ultramicrotome (RMC Boeckeler) (Jumbo 35° diamond knife (Diatome, Switzerland)). For further analysis, sections were transferred onto silicon wafer substrates. For post-staining the substrates (with sections facing down), the sections were placed on a 200 μL droplet of 3% uranyl acetate (Science Services, Cat# E22400) in water for 10 min, washed with water, and then immersed in a solution of 3% lead citrate (Science Services, Cat# DM22410) in water for 5 min.

#### Electron microscopy and image processing

Sections were imaged using a field emission scanning electron microscope (Ultra 55, Carl Zeiss Microscopy) operated at a primary electron energy of 1.5 keV. Secondary electron (SE2) and backscattered electron (ESB) detectors were used to capture images. Using the Atlas 5 software (Carl Zeiss Microscopy), automated acquisition of large scan fields was performed. The resulting image stacks were aligned and processed using the TrakEM module in Fiji.^38^

z stack of ultrastructural images (6 nm pixel size in x-y and 100 nm in z) was cropped and reduced in scale using Fiji and contrast was enhanced automatically. Segmentation of cells was done using Amira software (Thermo Fisher Scientific) (voxel size of 1-2-8.3). Cells within the cell cluster were outlined separately in x-y plane by using the segmentation tool and interpolated in z-plane. The segmented labels were visualized using the Volren display option.

### Quantification and statistical analysis

#### Quantification of retinal cell types in clusters

Cell type ratios and cell numbers within late retinal organoids were quantified manually from confocal imaging data using Fiji. The number of cells within a cluster was counted by using the cell counter tool. A cluster was defined as a group of Otx2-positive cells with two or more cells, which are located in close vicinity. Counting cell types within clusters was done using the cell counter tool and scoring the double-positive cells for the respective cell type specific label (Rx2-Otx2 – PR; Zpr1-Otx2 – cone PR) within an Otx2-positive cell cluster. Otx2-only cells in the Rx2-Otx2-double labeling were counted as BP cells. Distance measurements between Otx2-clusters was done by defining the center of each cluster in a defined z stack (sub-stack; up to 30 clusters per sub-stack) and documenting the position in 3D by recording the coordinates (measuring tool) using Fiji (ImageJ2). To define the distance between all clusters of a sub-stack, the vector length between all coordinates within one sub-stack were calculated (*n* = 159 clusters in *n* = 10 organoids; 2–3 sub-stacks per organoid). The datasets (‘all distances’) include the distances between not directly neighboring clusters, which were removed as outliers. To filter for the distances of direct neighboring clusters only (remove outliers), a range of values was defined by the average and 3× standard variation (±) of the smallest distance value within the distance data of each sub-stack. This range was used as boundaries within the ‘all distances’ data and the values were displayed using a kde (kernel density estimate) plot. Kde is a nonparametric distribution model and no underlying data distribution is assumed. Graphs were plotted and statistical analysis done in Python (Jupyter lab interface). N numbers for each quantification are noted in the respective figure legends.

## References

[1] Gehring W.J. (2014). The evolution of vision. WIREs Dev. Biol..

[2] Vopalensky P., Kozmik Z. (2009). Eye evolution: Common use and independent recruitment of genetic components. Philos. Trans. R. Soc. Lond. B Biol. Sci..

[3] Hahn J., Monavarfeshani A., Qiao M., Kao A.H., Kölsch Y., Kumar A., Kunze V.P., Rasys A.M., Richardson R., Wekselblatt J.B. (2023). Evolution of neuronal cell classes and types in the vertebrate retina. Nature.

[4] Lamb T.D., Collin S.P., Pugh E.N. (2007). Evolution of the vertebrate eye: Opsins, photoreceptors, retina and eye cup. Nat. Rev. Neurosci..

[5] Schwab I.R. (2018). The evolution of eyes: Major steps. the Keeler lecture 2017: Centenary of Keeler Ltd. Eye (Basingstoke).

[6] Arendt D., Wittbrodt J. (2001). Reconstructing the eyes of Urbilateria. Philos. Trans. R. Soc. Lond. B Biol. Sci..

[7] Weasner B.M., Kumar J.P. (2022). The timing of cell fate decisions is crucial for initiating pattern formation in the Drosophila eye. Development.

[8] Randlett O., Norden C., Harris W.A. (2011). The vertebrate retina: A model for neuronal polarization in vivo. Dev. Neurobiol..

[9] Das T., Payer B., Cayouette M., Harris W.A. (2003). In vivo time-lapse imaging of cell divisions during neurogenesis in the developing zebrafish retina. Neuron.

[10] Kitambi S.S., Malicki J.J. (2008). Spatiotemporal features of neurogenesis in the retina of medaka, Oryzias latipes. Dev. Dyn..

[11] Malicki J., Driever W. (1999). oko meduzy mutations affect neuronal patterning in the zebrafish retina and reveal cell-cell interactions of the retinal neuroepithelial sheet. Development.

[12] Pujic Z., Malicki J. (2001). Mutation of the Zebrafish glass onion Locus Causes Early Cell-Nonautonomous Loss of Neuroepithelial Integrity Followed by Severe Neuronal Patterning Defects in the Retina. Dev. Biol..

[13] Wei X., Malicki J. (2002). nagie oko, encoding a MAGUK-family protein, is essential for cellular patterning of the retina. Nat. Genet..

[14] Aparicio G., Rodao M., Badano J.L., Zolessi F.R. (2021). Photoreceptor progenitor dynamics in the zebrafish embryo retina and its modulation by primary cilia and N-cadherin. Int. J. Dev. Biol..

[15] Moscona A. (1961). Rotation-mediated histogenetic aggregation of dissociated cells: A quantifiable approach to cell interactions in vitro. Exp. Cell Res..

[16] Layer P.G., Willbold E. (1994). Regeneration of the avian retina by retinospheroid technology. Prog. Retin. Eye Res..

[17] Zilova L., Weinhardt V., Tavhelidse T., Schlagheck C., Thumberger T., Wittbrodt J. (2021). Fish primary embryonic pluripotent cells assemble into retinal tissue mirroring in vivo early eye development. eLife.

[18] Iwamatsu T. (2004). Stages of normal development in the medaka Oryzias latipes. Mech. Dev..

[19] Hu M., Easter S.S. (1999). Retinal neurogenesis: the formation of the initial central patch of postmitotic cells. Dev. Biol..

[20] Del Bene F., Ettwiller L., Skowronska-Krawczyk D., Baier H., Matter J.-M., Birney E., Wittbrodt J. (2007). In vivo validation of a computationally predicted conserved Ath5 target gene set. PLoS Genet..

[21] Dyer M.A., Livesey F.J., Cepko C.L., Oliver G. (2003). Prox1 function controls progenitor cell proliferation and horizontal cell genesis in the mammalian retina. Nat. Genet..

[22] Greferath U., Grünert U., Wässle H. (1990). Rod bipolar cells in the mammalian retina show protein kinase C-like immunoreactivity. J. Comp. Neurol..

[23] Crespo C., Knust E. (2018). Characterisation of maturation of photoreceptor cell subtypes during zebrafish retinal development. Biol. Open.

[24] Avanesov A., Malicki J. (2010). Analysis of the retina in the zebrafish model. Methods Cell Biol..

[25] Livesey F.J., Cepko C.L. (2001). Vertebrate neural cell-fate determination: Lessons from the retina. Nat. Rev. Neurosci..

[26] Almeida A.D., Boije H., Chow R.W., He J., Tham J., Suzuki S.C., Harris W.A. (2014). Spectrum of Fates: A new approach to the study of the developing zebrafish retina. Development.

[27] He J., Zhang G., Almeida A.D., Cayouette M., Simons B.D., Harris W.A. (2012). How variable clones build an invariant retina. Neuron.

[28] Centanin L., Hoeckendorf B., Wittbrodt J. (2011). Fate restriction and multipotency in retinal stem cells. Cell Stem Cell.

[29] Prieto-López L., Pereiro X., Vecino E. (2024). The mechanics of the retina: Müller glia role on retinal extracellular matrix and modelling. Front. Med..

[30] Dorgau B., Felemban M., Sharpe A., Bauer R., Hallam D., Steel D.H., Lindsay S., Mellough C., Lako M. (2018). Laminin γ3 plays an important role in retinal lamination, photoreceptor organisation and ganglion cell differentiation. Cell Death Dis..

[31] Eiraku M., Sasai Y. (2011). Mouse embryonic stem cell culture for generation of three-dimensional retinal and cortical tissues. Nat. Protoc..

[32] Nakano T., Ando S., Takata N., Kawada M., Muguruma K., Sekiguchi K., Saito K., Yonemura S., Eiraku M., Sasai Y. (2012). Self-formation of optic cups and storable stratified neural retina from human ESCs. Cell Stem Cell.

[33] Icha J., Kunath C., Rocha-Martins M., Norden C. (2016). Independent modes of ganglion cell translocation ensure correct lamination of the zebrafish retina. J. Cell Biol..

[34] Warren J., Kumar J.P. (2023). Patterning of the Drosophila retina by the morphogenetic furrow. Front. Cell Dev. Biol..

[35] Reinhardt R., Centanin L., Tavhelidse T., Inoue D., Wittbrodt B., Concordet J.P., Martinez-Morales J.R., Wittbrodt J. (2015). Sox2, Tlx, Gli3, and Her9 converge on Rx2 to define retinal stem cells in vivo. EMBO J..

[36] Loosli F., Köster R.W., Carl M., Kühnlein R., Henrich T., Mücke M., Krone A., Wittbrodt J. (2000). A genetic screen for mutations affecting embryonic development in medaka fish ( Oryzias latipes ). Mech. Dev..

[37] Zhu X., Huang L., Zheng Y., Song Y., Xu Q., Wang J., Si K., Duan S., Gong W. (2019). Ultrafast optical clearing method for three-dimensional imaging with cellular resolution. Proc. Natl. Acad. Sci. USA.

[38] Cardona A., Saalfeld S., Schindelin J., Arganda-Carreras I., Preibisch S., Longair M., Tomancak P., Hartenstein V., Douglas R.J. (2012). TrakEM2 software for neural circuit reconstruction. PLoS One.

