# Supplementary material for: Extrinsic polarity cues control lamination versus cluster-based organization in vertebrate retinal development
Source: iScience. 2026 Apr 11;29(5):115684. doi: 10.1016/j.isci.2026.115684 (PMC13138029; doi:10.1016/j.isci.2026.115684)
Supplement: Document S1. Figures S1–S6 [file mmc1.pdf]

iScience, Volume *29*

Supplemental information

Extrinsic polarity cues control lamination

# versus cluster-based organization

in vertebrate retinal development

Christina Schlagheck, Xenia Podlipensky, Cassian Afting, Ronald Curticean, Irene Wacker, Rasmus R. Schröder, Venera Weinhardt, Lucie Zilova, and Joachim Wittbrodt

Supplemental information

Document S1. Figures S1-6.

Supplemental figures

A

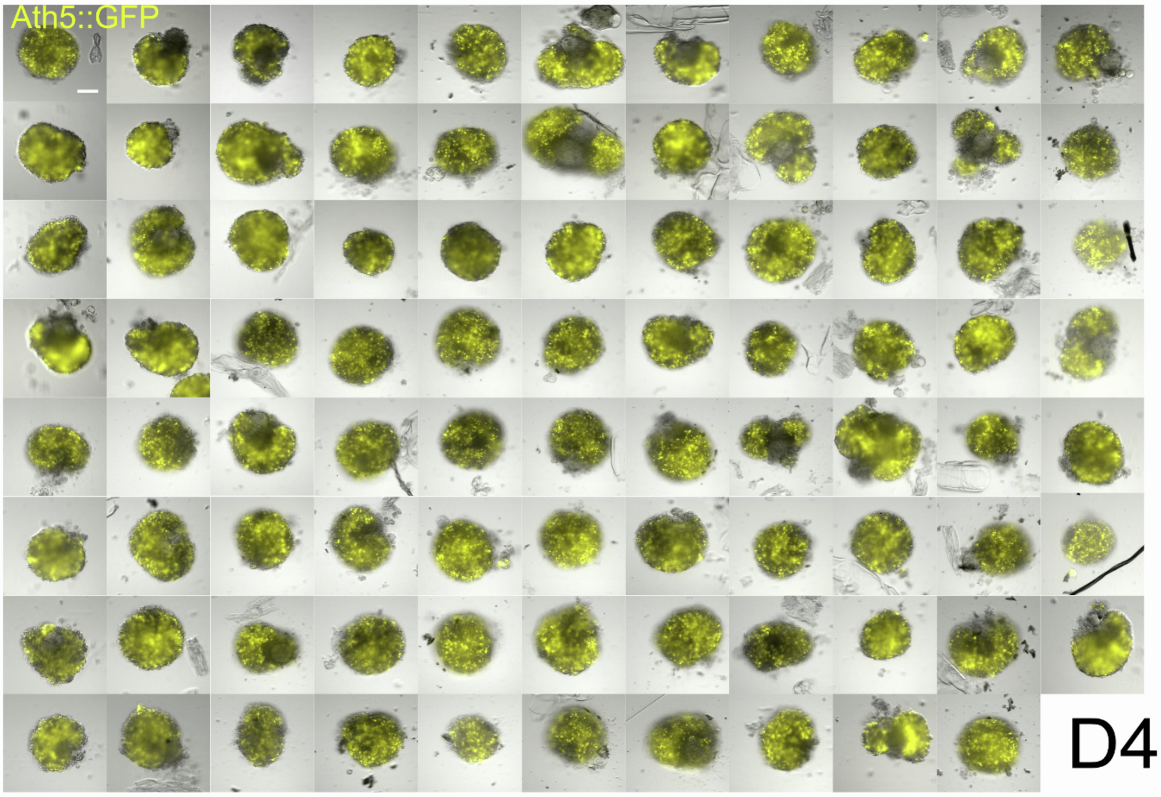

B

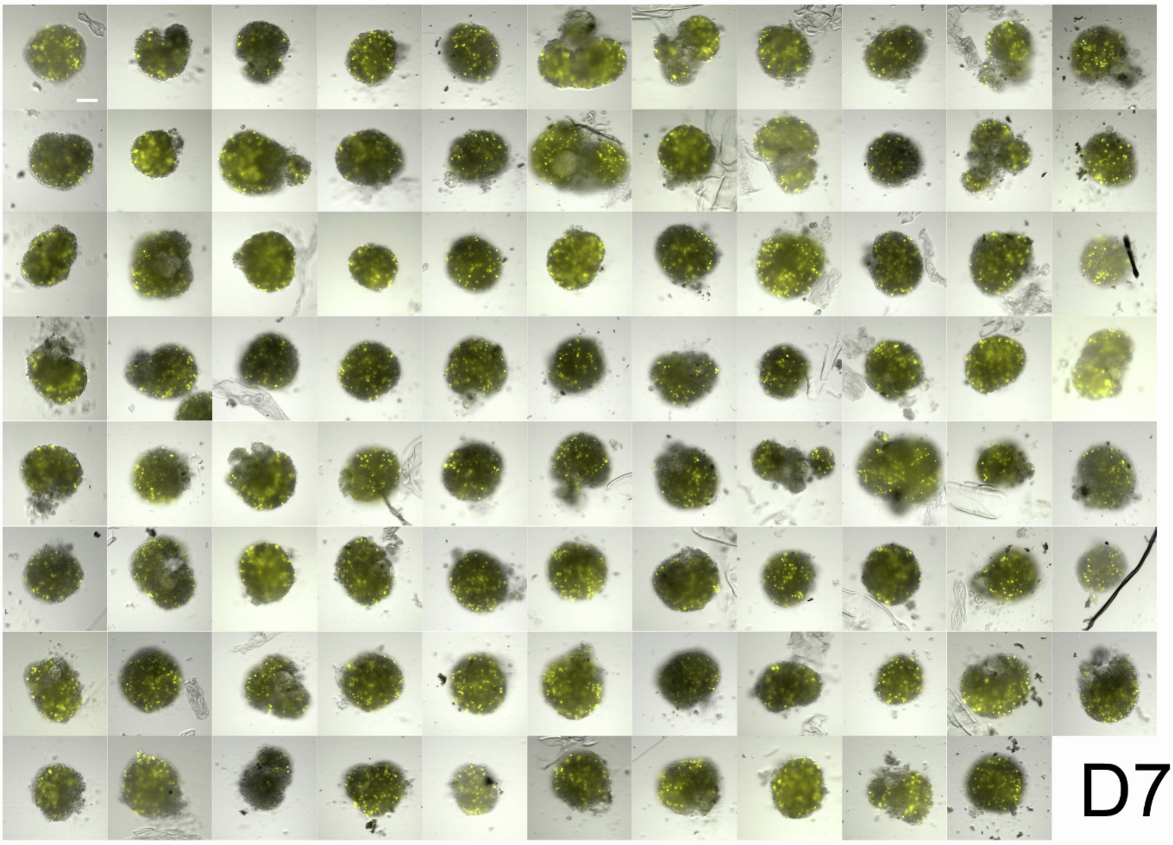

Figure S1: Differentiation of medaka retinal organoids containing RGCs is robust and reproducible, related to Figure 1.

Retinal organoids grown on one 96 well plate imaged with the Acquifer imaging machine on day four (A) and day seven (B). GFP signal of Ath5::GFP reporter shown in overlay with brightfield images. Scale bar 100 µm.

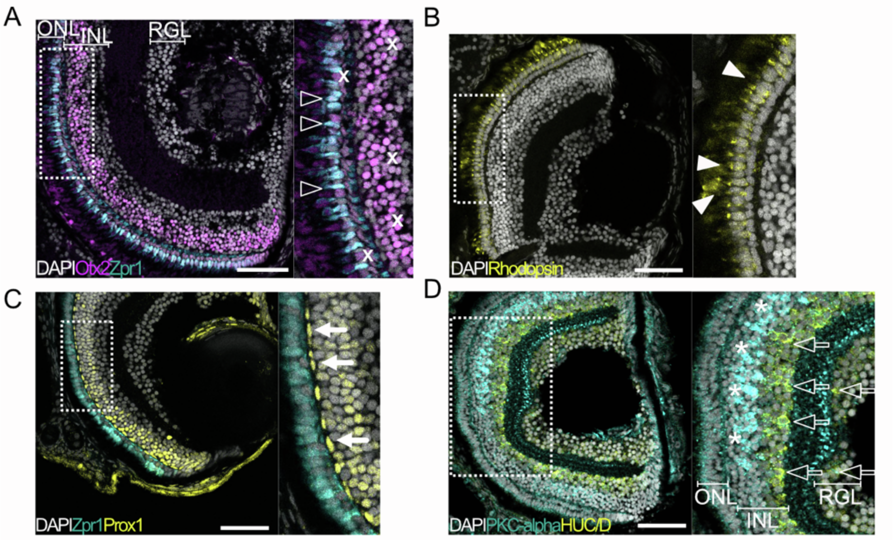

Figure S2: Retinal cell type distribution in embryonic retina of medaka, related to Figure 2.

Retinal cell types labeled with cell type-specific antibodies and DAPI-labeled nuclei (white) in sectioned fish retina at day 8 (0 days post hatch). ONL = outer nuclear layer; INL = inner nuclear layer; RGL = retinal ganglion cell layer. Scale bar 50 µm.

A) Otx2 antibody (magenta) labels photoreceptors (PRs) and bipolar cells (BPs) (both labeled by white x), Zpr1 (cyan) and Otx2 double immunolabeling showing cone PR (marked by unfilled arrow heads).

B) Rhodopsin (yellow) immunolabeling marking rod PR (marked by filled arrow heads).

C) Prox1 (yellow) immunolabeling marking horizontal cells (marked by filled arrows) adjacent to Zpr1-positive (cyan) cone PR.

D) PKC-alpha (cyan) antibodies labeling BP (marked by asterisk) and HuC/D (yellow) marking amacrine cells and retinal ganglion cells (RGCs) (both marked by unfilled arrows).

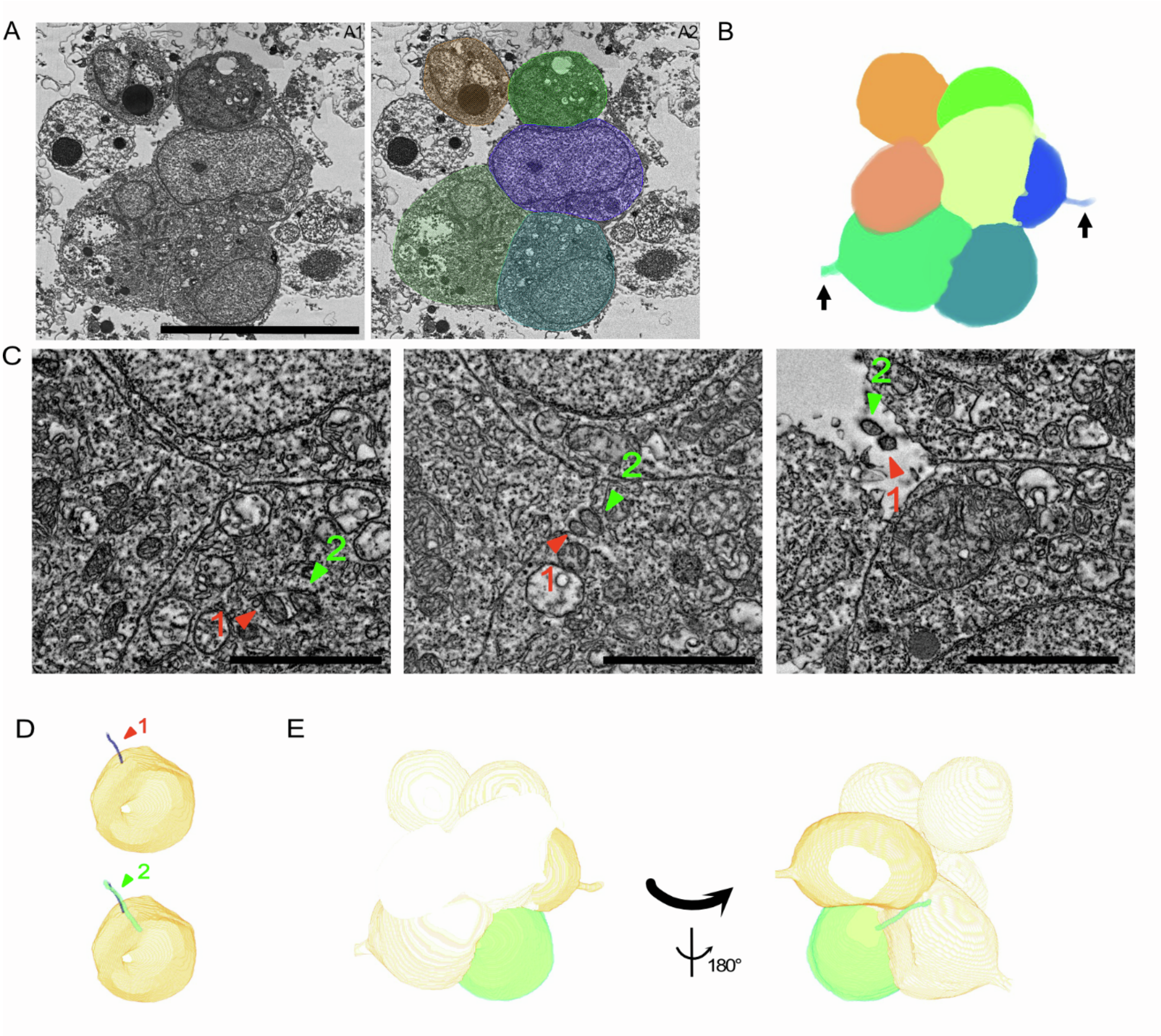

Figure S3: Ultrastructure of retinal cell clusters in medaka retinal organoids show tight association of cells and cells' cilia, related to Figure 2.

(A) Example of cell cluster in day seven medaka retinal organoid imaged with electron microscopy. Single z-plane of cell cluster showing five of seven tightly associated cells within the cluster. The same plane is overlayed with a segmentation markup using Amira. Scale bar 10µm.

(B) 3D projection of seven segmented cells forming a cluster. Three cells show axonal projections. Two of those are visible in the displayed orientation (indicated by arrows).

(C) Cell with two cilia projecting into the same direction and out of the cell. Cilia grow out along neighboring cells. Cilia marked by arrows and numbers. Scale bar 2µm.

(D) 3D volume of segmented cell in (C) with cilium1 only (top) and with both cilia (bottom).

(E) Cell with cilia (green) in context with the whole cell cluster shown in cell segmentation. Shown in the same orientation as the single cell display in (D) and in context as in (B) and turned by 180° on the vertical axis.

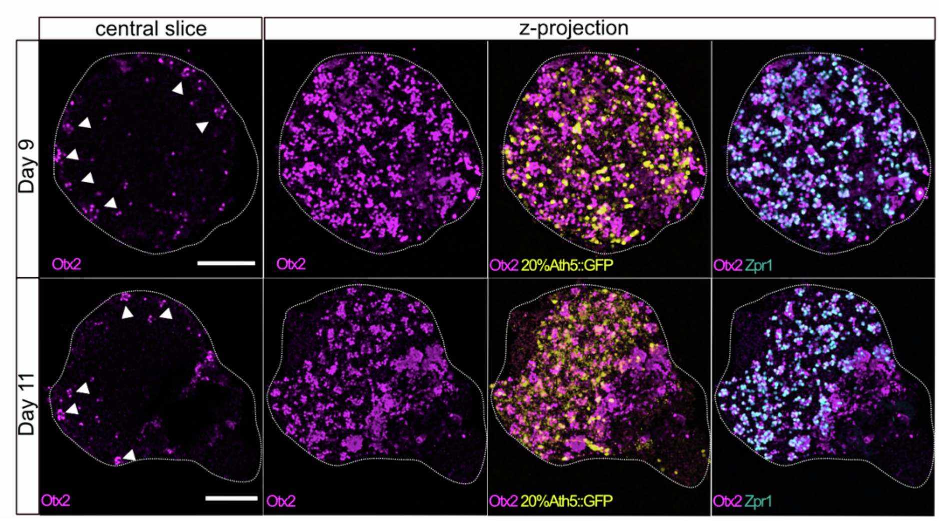

Figure S4: Retinal clusters in medaka retinal organoids are stable until at least day eleven, related to Figure 3.

Medaka retinal organoids derived of wild type (wt) and Ath5::GFP reporter cells (4:1) stained for the presence of Otx2, Zpr1 and GFP by immunohistochemistry on day 9 (n=11 organoids in three independent experiments) and day eleven (n=15 organoids in three independent experiments). Otx2 expressing cell clusters are indicated by white arrow heads. Scale bar 100 µm.

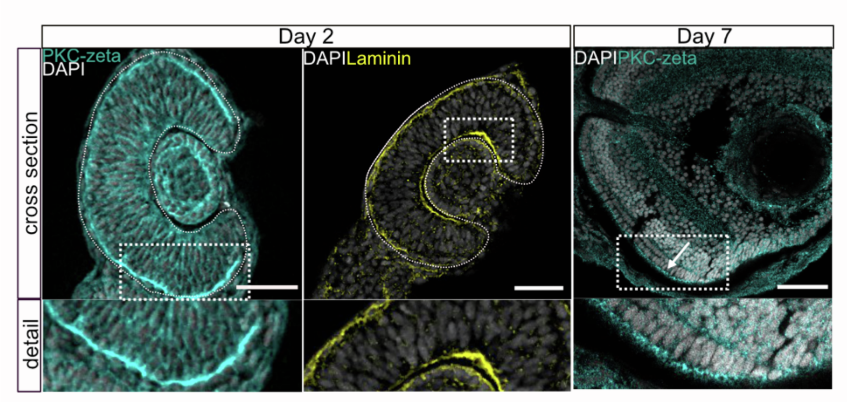

Figure S5: Medaka retina polarity, related to Figure 3.

Polarity of retinal tissue in medaka embryos on day two and day seven. Optical cross sections of embryonic retinae are shown. PKC-zeta marking apical cell polarity and Laminin accumulating at the basal border of the epithelium. The apical pole of the retinal tissue within the optic cup is facing the brain, while the basal side of the tissue is facing the lens. At day seven, the apical polarity in PR is still present at the interface to the RPE. Scale bar 50µm.

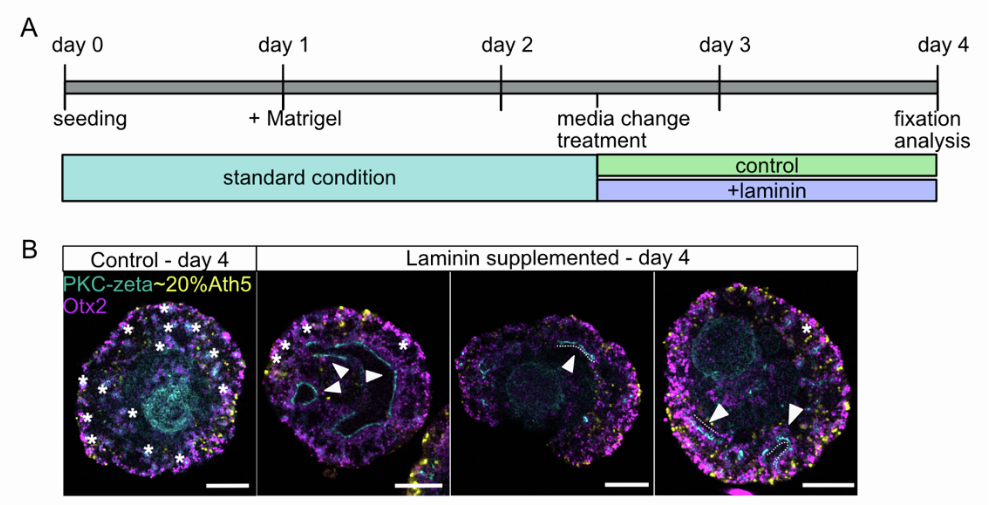

Figure S6: Phenotypic spectrum of medaka retinal organoids grown in laminin-supplemented culture, related to Figure 4.

(A) Experimental procedure for laminin-supplemented organoid culture. After culturing organoids in standard conditions until late day two, laminin was added to the 'treated' organoid. Samples were cultured until day four and then processed for antibody staining.

(B) Tissue structure in standard and laminin-supplemented culture. By day four, tissue stretches remain polarized, visible by Otx2-positive cells (magenta) lining up with the apical side (PKC-zeta, cyan) towards the core of the organoid (marked by arrows). Clusters can be found in other areas of the laminin-enriched organoids (marked by stars). Control organoids exclusively show clustered phenotype (marked by stars). Ath5::GFP marks a fraction (~20%) of RGCs and their distribution. Scale bar 100 µm.
